# Applicability of the Global Lung Initiative 2012 Reference Values for Spirometry for Longitudinal Data of Elderly Women

**DOI:** 10.1371/journal.pone.0157569

**Published:** 2016-06-16

**Authors:** Anke Hüls, Ursula Krämer, Sabine Stolz, Frauke Hennig, Barbara Hoffmann, Katja Ickstadt, Andrea Vierkötter, Tamara Schikowski

**Affiliations:** 1 IUF-Leibniz Research Institute for Environmental Medicine, Düsseldorf, Germany; 2 Medical Faculty, Deanery of Medicine, Heinrich-Heine University of Düsseldorf, Düsseldorf, Germany; 3 Faculty of Statistics, TU Dortmund University, Dortmund, Germany; Lee Kong Chian School of Medicine, SINGAPORE

## Abstract

**Background and Objectives:**

Lung function depends nonlinearly on age and height, so that the use of age and height specific reference values is required. The widely used age and height specific GLI (Global Lung Initiative) z-scores derived from cross-sectional data, however, have not been proven for validity in an elderly population or for longitudinal data. Therefore, we aimed to test their validity in a population of elderly women followed prospectively for more than 20 years.

**Methods:**

We used spirometric data (forced expiratory volume in 1 second (FEV_1_) and forced vital capacity (FVC) and FEV_1_/FVC) from the SALIA cohort of German women (baseline: 1985–1994 (aged 55 years), follow-up: 2008/2009 and 2012/2013). We calculated GLI-z-scores for baseline and follow-up examination separately (cross-sectional evaluation) and individual differences in z-scores between baseline and follow-up (longitudinal evaluation) for healthy never-smoking women.

**Results:**

GLI reference values for FEV_1_, FVC and FEV_1_/FVC were cross-sectionally and longitudinally equivalent with our SALIA data. The mean change in z-scores between baseline and follow-up was 0.33 for FEV_1_, 0.38 for FVC and -0.10 for FEV_1_/FVC.

**Conclusions:**

In conclusion, GLI z-scores fit cross-sectionally and longitudinally with FEV_1_, FVC and FEV_1_/FVC measured in women from Germany which indicates that they can be used in longitudinal association analyses.

## Introduction

Lung function depends nonlinearly on age and height [1–3]. Therefore, age and height specific reference values should be used to account for these dependencies in epidemiological data analysis.

In 2012, the Global Lung Initiative (GLI) developed new multi-ethnic spirometric reference values for the age range 3 to 95 years [4]. The GLI reference values consider a nonlinear age- and a linear height-dependency of lung function. However, the cross-sectional fit of the GLI reference values for subjects aged >75 years is not clear [4], because most of the included datasets consisted of children, adolescents or young adults and only few studies contributed to the derivation of reference values contained subjects above 75 years [5]. It is therefore necessary to investigate the validity of the GLI reference values for older populations.

In addition, the GLI reference values were derived from cross-sectional data and application on longitudinal data has not been evaluated. If a longitudinal validity is given, GLI reference values could provide a new option to make longitudinal change of lung function comparable between different age groups and thereby substantially enhance epidemiological analysis for respiratory risk factors. Furthermore, the use of reference values make it possible to evaluate whether the change of lung function deviates from the normal age-related decline in lung function.

We first aimed to analyse whether the newly developed GLI reference values fit cross-sectionally in a population of elderly women and compare their fit with older reference values. Secondly, we aimed to evaluate whether the GLI reference values can be used to describe longitudinal change in lung function. For the following analysis we used data from the SALIA study (Study on the influence of Air pollution on Lung function, Inflammation and Aging), a cohort of middle-aged women at baseline that was followed for more than 20 years [6].

## Material and Methods

### Study design and population

**Fig 1:**
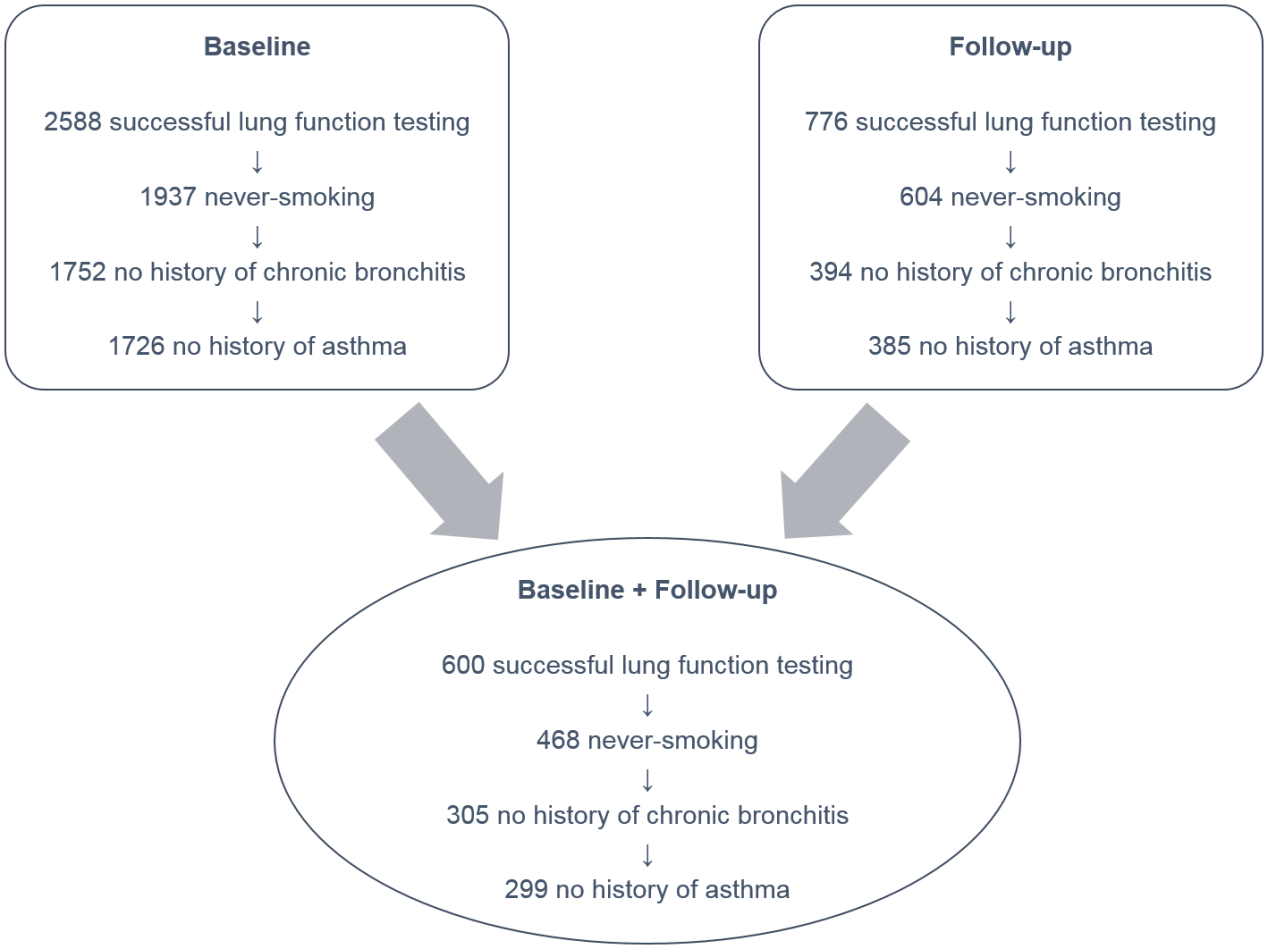
The SALIA collective at baseline (1985–1994) and follow-up examination (2008/2009 and 2012/2013).

A detailed description of the study population including detailed information about the respiratory health has been published previously [6–9]. Briefly, the Caucasian SALIA cohort study was initiated in the early 1980s to investigate the health effects of air pollution exposure in women. The study population consists of women, living in the industrialized Ruhr area and in the rural Southern Muensterland in Germany. Baseline examinations were conducted between 1985 and 1994 including 2588 women with successful lung function testing (aged 55 years) (Fig 1). The follow-up examinations took place in 2008/2009 and in 2012/2013 [6,7]. The following analysis included women who had at least one follow-up using the data for the one most remote from baseline. Furthermore the analysis was restricted to the healthy (no asthma or chronic bronchitis ever diagnosed regarding to the questionnaire answered by the participants) never-smoking women (HNSW). In total, the cross-sectional evaluation of the GLI reference values was restricted to 1726 women at baseline and 385 at follow-up. The longitudinal evaluation of the GLI reference values was based on the HNSW with lung function data at baseline and at least one follow-up (n = 299). Approval of the study was obtained from the Ethical Committee of the University of Bochum and the University of Düsseldorf. We received written informed consent from all participants [6].

### Lung function measurement

Forced expiratory volume in 1 second (FEV_1_) and forced vital capacity (FVC) were measured via spirometry. The values used in the analysis of this paper were all transformed to MasterScope Jaeger spirometer values because most investigations were performed with that device (37.54% of the baseline investigations and 81.56% of the follow-up investigations). Detailed information on the transformation equations is available in S1 Methods.

All devices were calibrated prior to testing. The technical personnel were trained and all results were reviewed by a pulmonary physician. Maximal expiration was intensively encouraged to achieve the best possible results, aiming for three technically acceptable spirometric manoeuvres in a maximum of nine trials. The best technically acceptable spirometric manoeuvre according to the ATS (American Thoracic Society) / ERS (European Respiratory Society) recommendations [10,11] including visual control [12] was chosen for analysis.

### Statistical methods

We first tested our data for a healthy survivor bias by comparing baseline lung function indices and baseline covariates of the HNSW lost to follow-up to baseline characteristics of the HNSW available at follow-up (two-sample t-test [13] and Fisher's exact test [14] at the 5% significance level).

To evaluate cross-sectional fit of the GLI reference values to the spirometric values of the HNSW we calculated the GLI-z-scores for baseline and follow-up examination. An absolute mean z-score > 0.5 was set as cut point for relevant differences to the GLI reference population (expected mean: 0) [15–17]. For a good cross-sectional fit, the mean should be approximately zero (mean within the interval [-0.5, 0.5]) at the 5% significance level (tested with two one-sided tests (TOST) for equivalence [13,18–20]). Furthermore, standard deviation and percentage below the lower limit of normal (LLN) were calculated.

In the HNSW with lung function measurements at baseline and follow-up examination the fit of the GLI reference values was graphically depicted and was additionally compared to the fit of the most common older reference values (NHANES III [21] and ECSC [22]).

Longitudinal fit of the GLI reference values was analysed in the HNSW with lung function measurements at baseline and follow-up examination using the subjects’ individual changes in lung function between baseline and follow-up (z_f_-z_b_). For a good longitudinal fit, these differences should be approximately zero (mean deviations within the interval [-0.5, 0.5]) at the 5% significance level (tested with the TOST for equivalence).

We performed three sensitivity analyses. In the first sensitivity analysis we evaluated cross-sectional fit of the GLI reference values in the HNSW who participated in the baseline and at least one follow-up examination. In a second sensitivity analysis we used a stricter definition of “healthy” and excluded additionally participants with symptoms of chronic bronchitis (cough and phlegm for ≥3 months of the year for ≥2 years), chronic cough and chronic phlegm. Furthermore, in a third sensitivity analysis we reduced our study population to the HNSW who performed the spirometric measurement with MasterScope Jaeger to validate our findings.

More detailed information on the statistical methods is available in S1 Methods.

All analyses were conducted using R 3.1.1 [23].

## Results

### Study population

Our study population consists of all HNSW with spirometric data at one or more examination times (baseline or at least one follow-up). The mean age was 54 years at baseline and 75 years at follow-up (Table 1). In an univariate analysis of the baseline characteristics, significant risk factors for a lost to follow-up were a high age and BMI, a low socio economic status, a low FEV_1_ and a low FVC at baseline (Table 2).

**Table 1:** Description of the study characteristics of the healthy never-smoking women (HNSW) at baseline and follow-up examination.

	Baseline	Follow-up
N	1726	385*
Age, mean (min-max)	54.46 (0.72)	75.47 (3.61)
BMI, mean (sd)	27.75 (4.69)	27.66 (4.39)
Socio economic status^†^, n (%)		
Low socio economic status^†^, n (%)	446 (25.84%)	62 (16.10%)
Medium socio economic status^†^, n (%)	855 (49.54%)	191 (49.61%)
High socio economic status^†^, n (%)	422 (24.45%)	130 (33.77%)
Passive smoking, n (%)	763 (44.21%)	77 (20.00%)

*: n = 222 from 2012/2013 and n = 163 from 2008/2009

^†^_:_ level of education asked at baseline

**Table 2:** Comparison of baseline characteristics of healthy never-smoking women (HNSW) lost to follow-up and available at follow-up. Differences in the continuous variables were tested with the two-sample t-test and differences in the categorical variables were tested with Fisher’s exact test at the 5% significance level.

	Baseline characteristics of those		
	Lost to follow-up*	Available at follow-up	p-value
N	1427	299	
mean age (sd)	54.49 (0.70)	54.30 (0.80)	<0.001^†^
mean BMI (sd)	27.97 (4.84)	26.69 (3.74)	<0.001
Low socio economic status^#^, n (%)	397 (27.82%)	49 (16.39%)	<0.001
Medium socio economic status^#^, n (%)	708 (49.61%)	147 (49.16%)	
High socio economic status^#^, n (%)	321 (22.49%)	101 (33.78%)	
Passive smoking, n (%)	644 (45.13%)	119 (39.80%)	0.095
mean z-score FEV_1_ (sd)	-0.46 (1.06)	-0.11 (0.90)	<0.001
%≤LLN z-score FEV_1_	12.89%	3.68%	
mean z-score FVC (sd)	-0.25 (0.99)	0.07 (0.81)	<0.001
%≤LLN z-score FVC	7.29%	2.34%	
mean z-score FEV1/FVC (sd)	-0.41 (0.88)	-0.35 (0.79)	0.301
%≤LLN z-score FEV_1_/FVC	8.13%	5.35%	

*: 748 women died between baseline and 2008/2009 and 37 women died between 2008/2009 and 2012/2013

^†^: only significant because all women entered the study population at the same age (extremely low sd)

^#^: level of education asked at baseline

### Cross-sectional fit of GLI reference values

There were no relevant differences between the GLI reference population and our HNSW for the mean z-scores for FEV_1_, FVC and FEV_1_/FVC at baseline and follow-up and the standard deviations were approximately one (Table 3 and S1 Fig). Equivalence between the GLI reference population and our HNSW was significant for all three lung function parameters at baseline and follow-up.

**Table 3:** GLI-based z-scores of all healthy never-smoking women (HNSW) grouped by time of examination (baseline and follow-up). Two one-sided tests for equivalence were performed to establish equivalence between the mean z-score of the GLI reference population and SALIA (p<0.05).

	N	Mean	sd	p-value*	%≤LLN
**Baseline**					
FEV_1_	1726	-0.40	1.04	<0.001	11.30
FVC	1724	-0.20	0.97	<0.001	6.50
FEV_1_/FVC	1722	-0.40	0.86	<0.001	7.67
**Follow-up**					
FEV_1_	385	0.23	1.03	<0.001	2.86
FVC	385	0.41	0.99	0.047	2.08
FEV_1_/FVC	385	-0.39	0.84	0.006	7.01

FEV_1_: forced expiratory volume in 1 s; FVC: forced vital capacity; LLN: Lower Limit of Normal.

*: Two one-sided tests (TOST) for equivalence were performed to test on equivalence between the mean z-scores of the GLI reference population and SALIA. A good fit was reached if the null-hypothesis of a mean z-score outside of the interval [-0.5, 0.5] was rejected at the 5% significance level (H_0_: zi−zj ∉ [−0.5,0.5]).

### Longitudinal fit of GLI reference values

**Fig 2:**
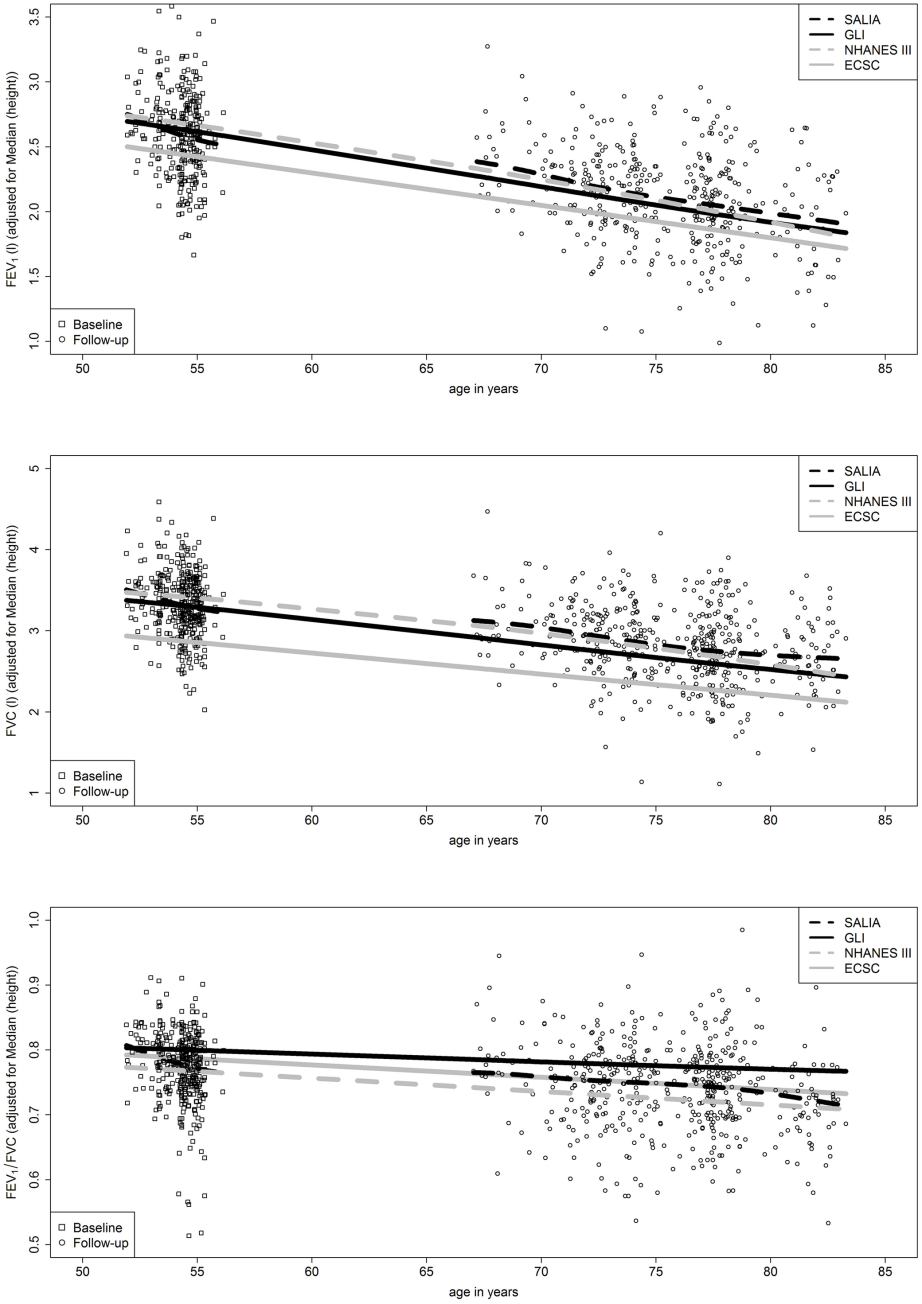
Comparison of measured SALIA values (HNSW) for FEV1, FVC and FEV1/FVC with height-adjusted predicted means of the GLI reference values and older NHANES III and ECSC reference values (n = 299).

For our study population, the predicted means of the GLI reference values were approximately linear with age (Fig 2). The age-related slope of the predicted means of the GLI reference values fitted well to the mean slope of FEV_1_ and FVC measured longitudinally in the HNSW over a period of more than 20 years. Furthermore, all mean changes of z-scores differed less than 0.5 from zero (p<0.01) which means that the longitudinal fit was good (Table 4).

**Table 4:** The longitudinal validity of the GLI reference values. Difference between z-scores at baseline and follow-up (z_f_ − z_b_). Two one-sided tests for equivalence were performed to establish equivalence between the mean z-scores of the GLI reference population and SALIA.

Follow-up—baseline	N	Mean	sd	p-value*
FEV_1_	299	0.33	0.80	<0.001
FVC	299	0.38	0.81	0.005
FEV_1_/FVC	299	-0.10	0.84	<0.001

FEV_1_: forced expiratory volume in 1 s; FVC: forced vital capacity; LLN: Lower Limit of Normal.

*: Two one-sided tests (TOST) for equivalence were performed to test on equivalence between the mean deviations in z-scores of the GLI reference population and SALIA. A good longitudinal fit was reached if the null-hypothesis of the mean deviations in z-scores outside of the interval [-0.5, 0.5] was rejected at the 5% significance level (H_0_: zi−zj ∉ [−0.5,0.5]).

### Sensitivity analyses

In a first sensitivity analysis we reduced our study population to the HNSW with lung function available at baseline and follow-up and evaluated again the cross-sectional fit. In this analysis we received similar results as in the main analysis, but the fit was not significant for FVC and FEV_1_/FVC at follow-up examination (S1 Table).

In a second sensitivity analysis we used a stricter definition of “healthy” and excluded additionally participants with symptoms of chronic bronchitis (cough and phlegm for ≥3 months of the year for ≥2 years), chronic cough and chronic phlegm. In this analysis we could confirm the cross-sectional and longitudinal fit of the GLI reference values (S2 and S3 Tables).

In a third sensitivity analysis we reduced our study population to the HNSW who performed the spirometric measurement with MasterScope Jaeger. In this analysis we received similar results as in the main analysis, but caused by the smaller study population the fit for FVC was not significant in the cross-sectional analysis at follow-up examination. The cross-sectional fit for FEV_1_ was better in this sub-sample leading to lower values for FEV_1_/FVC at follow-up examination (S4 Table). The longitudinal fit was again confirmed for all lung function measurements (S5 Table).

### Comparison with older reference values

The predicted GLI reference values for FEV_1_ and FVC differed only slightly from the NHANES III reference values [21] in level and slope (Fig 2). The slope of the predicted means of the ECSC reference values [22] was also similar to the slope of the predicted means of the GLI reference values, but the ECSC reference values for FEV_1_ and FVC were lower than GLI and NHANES III reference values and underestimated FEV_1_ and FVC measured in our HNSW. The level of the predicted means of GLI and NHANES III fitted well to the mean level of FEV_1_ and FVC measured in the HNSW with an even better fit for the NHANES III values. For FEV_1_/FVC the level of the predicted means of ECSC which lay between the level of GLI and NHANES III fitted best to FEV_1_/FVC measured in the HNSW, whereas GLI slightly overestimated FEV_1_/FVC.

## Discussion

GLI reference values provide a good cross-sectional and longitudinal fit with FEV_1_, FVC and FEV_1_/FVC measured over a period of more than 20 years in German women aged 52–83 years and can be used in longitudinal association analyses.

### Cross-sectional fit of GLI reference values

The GLI reference values for FEV_1_, FVC and FEV_1_/FVC provided a good cross-sectional fit in the SALIA population for baseline and follow-up. A good cross-sectional fit of the GLI reference values was also determined by Hall et al. (2012) in 2066 Caucasian subjects aged 4–80 years from Australia and New Zealand [15] and by Backman et al. (2015) in 501 Caucasian subjects (244 women) aged 22–91 years from Sweden [24]. Contrary to those studies we focused especially on the fit in the elderly (67–83 years of age at follow-up) which was unsure according to Quanjer et al. (2012) [4] who developed the GLI reference equations. Our study showed that the GLI reference values for FEV_1_, FVC and FEV_1_/FVC were also applicable for this age group. However, Miller et al. showed in their study of 592 Danes born in 1905 (428 females, mean age 93 years), that in a very elderly not selected population the GLI reference values might overestimate FEV_1_ because the GLI reference values for this subgroup are based on a select “supranormal” group of survivors who are functionally and cognitively inclined to participate [25]. In the SALIA study we also observed that the GLI reference values slightly overestimated FEV_1_ at baseline, whereas the fit was almost perfect for the healthy never-smoking participants who participated at baseline and follow-up investigation. This observation confirmed the findings of Miller et al. (2014) because our study population at baseline was less selected than at follow-up.

### Longitudinal fit of GLI reference values

The GLI reference values for FEV_1_, FVC and FEV_1_/FVC provided a good fit in level and age-related slope in our SALIA population and could consequently be used in a longitudinal analysis of the change in lung function over time.

Until now, the common opinion was that cross-sectional reference values are often not suitable for longitudinal data because cross-sectional data might be affected by cohort effects [1,26,27]. As longitudinal values are measured in the same subjects (often from the same generation) who are examined several times over a long time period there might be a difference to cross-sectional reference values which are measured in subjects from different generations. The majority of the GLI reference studies were carried out in the 90s [5] which is earlier than our follow-up examinations which were conducted in 2008/2009 and 2012/2013. Therefore the SALIA data are from a later cohort of people older than 70 years than those included in the reference values. Due to improvements in living conditions and a further enhancement of the medical care, we assume that if there were cohort effects in the GLI reference values, the follow-up measurements of the SALIA study would be higher than predicted by the GLI. However, the mean z-scores in this cohort increase only slightly with age.

### Comparison with the older ECSC and NHANES III reference values

The ECSC reference values [22] for FEV_1_ and FVC were much lower than GLI / NHANES III reference values and did not fit to the SALIA participants, which is consistent with results from previous studies [24,28,29]. However, for FEV_1_/FVC the ECSC reference values fitted well with our HNSW. This is in line with Kainu et al. (2015) who observed the same in their analysis of Finish adults (n = 1000, age: 18–83) [30].

The predicted means of the NHANES III reference values for FEV_1_ and FVC [21] were very similar to the predicted means of the GLI reference values which was already reported in Backman et al. (2015) [24]. Furthermore, the match between the NHANES III reference values for FEV_1_ and FVC and the healthy SALIA women was almost perfect. This is in line with Miller et al. (2014) in which the NHANES III equations performed the best [25]. In contrast, in a previous comparison study of 1302 healthy 20-80-year-old Germans the NHANES III reference values overestimated FVC [28]. However, since in that study 4.1% of the women had a lung function below the LLN for FEV_1_ and 7.5% below the LLN for FVC, we consider this over-estimation not to be physiologically relevant.

The age-related slopes of both ECSC and NHANES III reference values were similar to the slope of the GLI reference values. Consequently, the NHANES III reference values can still be used for Caucasian women older than 52 years. However, for men or other age groups the differences between NHANES III and GLI reference values might be larger.

### Strengths and limitations

Since research on lung function of subjects older than 75 years is limited, our SALIA study with a mean age of 75 years at follow-up provides important results on change in lung function in the elderly. Furthermore, to our knowledge this is the first study that indicates that the longitudinal change of lung function can be evaluated by using GLI-z-scores which offers a good opportunity for a standardized evaluation of longitudinal lung function data in clinical practice as well as in epidemiological research.

One limitation of the SALIA cohort is a selection towards healthy and surviving participants during the study duration of more than 20 years. Since respiratory health was a predictor for cardiovascular mortality in the SALIA cohort [31], we saw an increase of z-scores for FEV_1_ and FVC from baseline to follow-up examination in the cross-sectional analysis. However, lung function measured in this subset of healthy survivors fits to the GLI reference values for all examination times without relevant deviations. The reason might be that there is also a healthy survivor bias in the cross-sectional GLI reference data which is in line with Miller et al. (2014) who declared that the GLI reference values for the very elderly were based on a subset of “supranormal” survivors [25]. Another limitation of our study is the use of different lung function measurement devices and the weakness of the re-calibration equations we used to control for that because our re-calibration equation used to make EasyOne-measurements comparable to Jaeger-measurements were derived from the data of only 28 subjects. However, since the EasyOne device was only used in 71 of the included follow-up investigations (18.44%) the possible device related bias might not have a notable impact on our results.

Furthermore, the fairly high cut point of 0.5 for a relevant mean difference to the GLI reference population which was suggested by the GLI and equates to a difference of ~6% predicted [15] needs to be further evaluated for its relevance in clinical medicine as well as in epidemiological studies.

In conclusion, GLI reference values provide a good cross-sectional and longitudinal fit regarding FEV_1_, FVC and FEV_1_/FVC measured in elderly women from Germany over a time period more than 20 years and can be used in longitudinal association analyses. However, a regular update of GLI reference values is necessary to avoid cohort effects in future analyses.

## Supporting Information

S1 Fig The cross-sectional validity of the GLI-z-scores for FEV_1_, FVC and FEV_1_/FVC for the healthy never-smoking SALIA women (HNSW). (PDF)

S1 Methods Lung function measurement and statistical methods. (PDF)

S1 Table GLI-based z-scores of all healthy never-smoking women (HNSW) with available lung function measurements at baseline and follow-up grouped by time of examination. Two one-sided tests for equivalence were performed to establish equivalence between the mean z-score of the GLI reference population and SALIA (p<0.05).
(PDF)

S2 Table GLI-based z-scores of all healthy never-smoking women (HNSW) without symptoms of chronic bronchitis (cough and phlegm for ≥3 months of the year for ≥2 years), chronic cough and chronic phlegm grouped by time of examination (baseline and follow-up). Two one-sided tests for equivalence were performed to establish equivalence between the mean z-score of the GLI reference population and SALIA (p<0.05).
(PDF)

S3 Table The longitudinal validity of the GLI reference values of all healthy never-smoking women (HNSW) without symptoms of chronic bronchitis (cough and phlegm for ≥3 months of the year for ≥2 years), chronic cough and chronic phlegm. Difference between z-scores at baseline and follow-up (z_f_ − z_b_). Two one-sided tests for equivalence were performed to establish equivalence between the mean z-scores of the GLI reference population and SALIA.
(PDF)

S4 Table GLI-based z-scores of all healthy never-smoking women (HNSW) who performed the spirometric measurement with MasterScope Jaeger grouped by time of examination (baseline and follow-up). Two one-sided tests for equivalence were performed to establish equivalence between the mean z-score of the GLI reference population and SALIA (p<0.05).
(PDF)

S5 Table The longitudinal validity of the GLI reference values of all healthy never-smoking women (HNSW) who performed the spirometric measurement with MasterScope Jaeger. Difference between z-scores at baseline and follow-up (z_f_ − z_b_). Two one-sided tests for equivalence were performed to establish equivalence between the mean z-scores of the GLI reference population and SALIA.
(PDF)

## Abbreviations

GLI: Global Lung Initiative
FEV_1_: forced expiratory volume in 1 second
FVC: forced vital capacity
SALIA: Study on the influence of Air pollution on Lung function, Inflammation and Aging
HNSW: healthy (no asthma or chronic bronchitis ever diagnosed regarding to the questionnaire answered by the participants) never-smoking women
ATS: American Thoracic Society
ERS: European Respiratory Society
LLN: lower limit of normal
