# Supplementary material for: Applicability of the Global Lung Initiative 2012 Reference Values for Spirometry for Longitudinal Data of Elderly Women
Source: PLoS One. 2016 Jun 16;11(6):e0157569. doi: 10.1371/journal.pone.0157569 (PMC4910985; doi:10.1371/journal.pone.0157569)
Supplement: S1 Fig — (PDF) [file pone.0157569.s001.pdf]

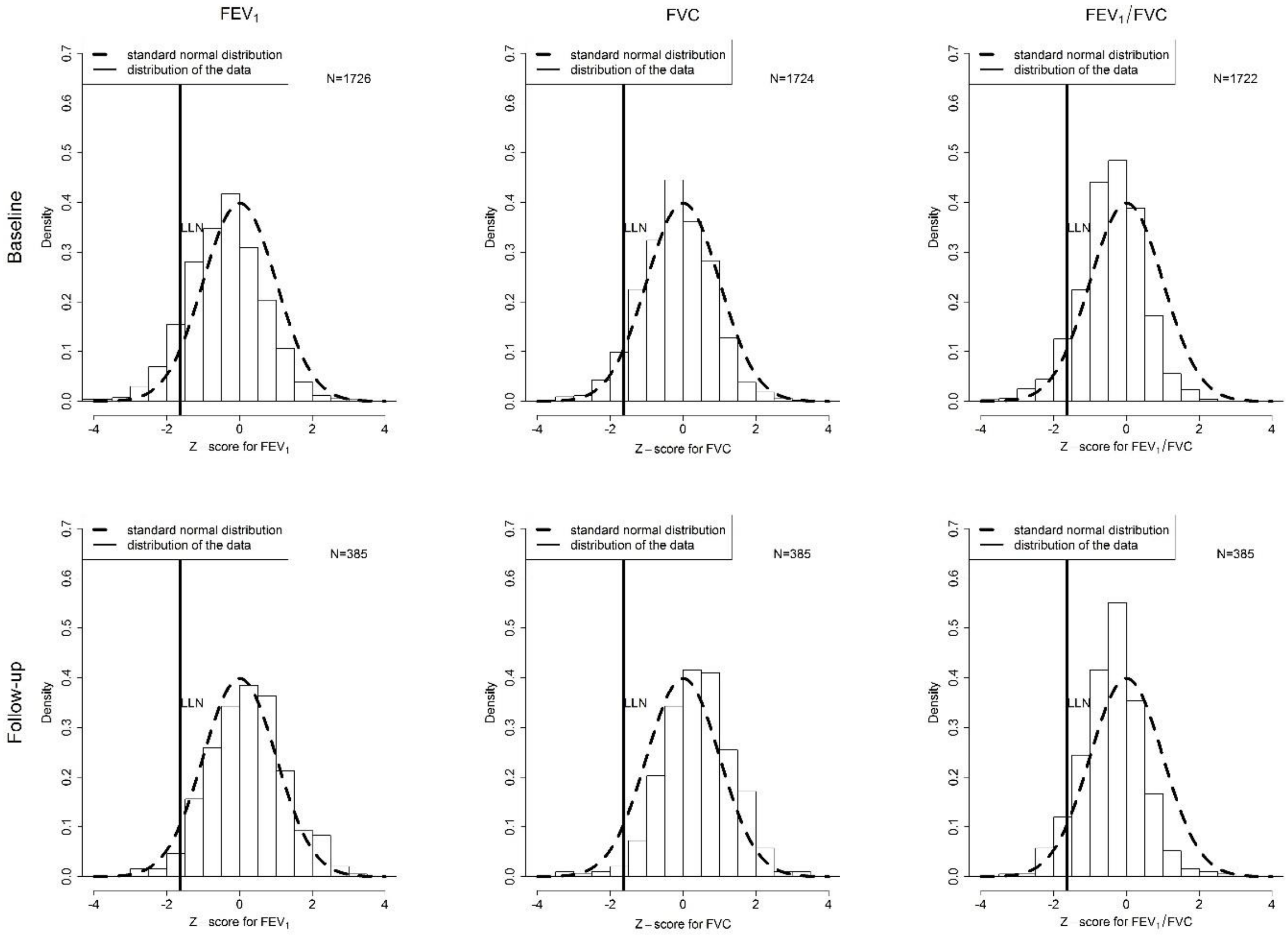


**S1 Fig: The cross-sectional validity of the GLI-z-scores for FEV1, FVC and FEV1/FVC for the healthy never-smoking SALIA women (HNSW).**
