# Supplementary material for: Applicability of the Global Lung Initiative 2012 Reference Values for Spirometry for Longitudinal Data of Elderly Women
Source: PLoS One. 2016 Jun 16;11(6):e0157569. doi: 10.1371/journal.pone.0157569 (PMC4910985; doi:10.1371/journal.pone.0157569)
Supplement: S1 Methods — (PDF) [file pone.0157569.s002.pdf]

S1 Methods

Lung function measurement

Forced expiratory volume in 1 second (FEV_1) and forced vital capacity (FVC) were measured via spirometry. For the baseline examination, initially the volume-displacement dry spirometer Vicatest 4 (Mijnhardt, Rotterdam, Holland) was used and later replaced with MasterScope Jaeger (Viasys Healthcare, Hoechberg) which is a heated handheld pneumotachograph. 116 women had measurements with both devices. Since we investigated differences between the two devices we established a regression equation from the double measurements for transforming the values between devices: $FEV_{1\ Jaeger} = 0.96216 * FEV_{1\ Vica} -0.01311$; $FVC_{Jaeger} = 1.037 * FVC_{Vica} – 0.01072$. In the second half of the first follow-up examination the MasterScope Jaeger was replaced with the EasyOne ultrasonic spirometer (ndd Medizintechnik, Zurich, Switzerland) which is a handheld spirometer that uses an ultrasonic sensor to measure air flow [1]. 28 participants were investigated with both devices and the following transformation equations were developed to correct for the differences between the devices: $FEV_{1\ Jaeger} = 1.03671 * FEV_{1\ NDD} + 0.21955$; $FVC_{Jaeger} = 1.10797 * FVC_{NDD} – 0.04149$ [2]. At second follow-up all women were investigated using the MasterScope Jaeger device because most baseline and follow-up measurements were performed with this device.

Statistical methods

Since our study was performed over a long period of time (1985-2013), we checked our dataset for a healthy survivor bias. Therefore, we compared baseline lung function indices and baseline covariates of the healthy never-smoking women (HNSW) lost to follow-up to baseline characteristics of the HNSW available at follow-up (two-sample t-test [3] and Fisher's exact test [4] at the 5% significance level.

We calculated GLI reference values for a subject's predicted mean and the corresponding GLI z-scores for FEV_1, FVC or FEV_1/FVC [5]. Since, the GLI reference values were developed on a healthy never-smoking reference population, we evaluated the fit of these values in the HNSW.

To evaluate cross-sectional fit of the GLI reference values to the spirometric values of the HNSW we calculated the GLI-z-scores for baseline and follow-up examination. Due to the expected standard normal distribution of the z-scores, the mean has to be zero for every age if the reference values fit to the analysed data. As was suggested by the GLI and was already done in other studies an absolute mean z-score > 0.5 was set as cut point for relevant differences to the GLI reference population [6–8]. Two one-sided tests (TOST) for equivalence [3,9–11] were performed to test the equivalence between the mean z-scores of the GLI reference population and SALIA. A good fit was reached if the null-hypothesis of a mean z-score outside of the interval [-0.5, 0.5] was rejected at the 5% significance level. Additional to the mean z-score, the standard deviation and the percentage below the lower limit of normal (LLN) were calculated for the HNSW. In clinical practice the LLN is often used to decide if a subject's lung function is 'abnormal'. Approximately 5% of the calculated z-scores should lay below the LLN of the GLI z-scores (LLN is given as -1.64).

In the HNSW with lung function measurements at baseline and follow-up examination the fit of the GLI reference values was graphically depicted and was additionally compared to the fit of the most common older reference values (NHANES III [12] and ECSC [13]) in different age groups. Repeated lung function measurements (adjusted for median height at baseline) of the HNSW were plotted against age together with an LMS regression curve with the height adjusted lung function as dependent variable and only age as explanatory variable. Additionally, an LMS regression curve of the GLI reference values for the predicted mean (adjusted for median height) and linear regression lines of the ECSC and NHANES III reference values (calculated for median height) were plotted against age.

The longitudinal fit of the GLI reference values was also analysed in the HNSW with lung function measurements at baseline and follow-up examination using the subjects' individual changes in lung function between baseline and follow-up. The distribution of the differences between the z-scores at baseline and follow-up (z_f-z_b) was analysed. For a good longitudinal fit, these differences should be approximately zero (mean deviations within the interval [-0.5, 0.5]) at the 5% significance level (tested with the TOST for equivalence).

In a sensitivity analysis we evaluated the cross-sectional fit of the GLI reference values in the HNSW who participated in the baseline and at least one follow-up examination.

All analyses were conducted using R 3.1.1 [14].

References

1. Walters J a E, Wood-Baker R, Walls J, Johns DP. Stability of the EasyOne ultrasonic spirometer for use in general practice. Respirology. 2006;11: 306–310. doi:10.1111/j.1440-1843.2006.00842.x

2. Adam M, Schikowski T, Carsin AE, Cai Y, Jacquemin B, Sanchez M, et al. Adult lung function and long-term air pollution exposure. ESCAPE: a multicentre cohort study and meta-analysis. Eur Respir J. 2015;45: 38–50.

3. Wellek S. Testing Statistical Hypotheses of Equivalence and Noninferiority. Second Edi. Crc Pr Inc; 2010.

4. Fisher RA. On the interpretation of χ2 from contingency tables, and the calculation of P. J R Stat Soc. 1922;85: 87–94.

5. Quanjer PH, Stanojevic S, Cole TJ, Baur X, Hall GL, Culver BH, et al. Multi-ethnic reference values for spirometry for the 3-95-yr age range: the global lung function 2012 equations. Eur Respir J. 2012;40: 1324–43.

6. Hall GL, Thompson BR, Stanojevic S, Abramson MJ, Beasley R, Coates A, et al. The Global Lung Initiative 2012 reference values reflect contemporary Australasian spirometry. Respirology. 2012;17: 1150–1.

7. Thompson BR, Stanojevic S, Abramson MJ, Beasley R, Coates A, Dent A, et al. The all-age spirometry reference ranges reflect contemporary Australasian spirometry. Respirology. 2011;16: 912–7.

8. Ben Saad H, El Attar MN, Hadj Mabrouk K, Ben Abdelaziz A, Abdelghani A, Bousarssar M, et al. The recent multi-ethnic global lung initiative 2012 (GLI2012) reference values don't reflect contemporary adult's North African spirometry. Respir Med. 2013;107: 2000–8.

9. Robinson AP, Froese RE. Model validation using equivalence tests. Ecol Modell. 2004;176: 349–358.

10. Schuirmann DL. On hypothesis testing to determine if the mean of a normal distribution is contained in a known interval. Biometrics. 1981;37: 617.

11. Westlake WJ. Response to T.B.L. Kirkwood: bioequivalence testing - a need to rethink. Biometrics. 1981;37: 589–594.

12. Hankinson JL, Odencrantz JR, Fedan KB. Spirometric reference values from a sample of the general U.S. population. Am J Respir Crit Care Med. 1999;159: 179–87. doi:10.1164/ajrccm.159.1.9712108

13. Quanjer PH, Tammeling GJ, Cotes JE, Pedersen OF, Peslin R, Yernault JC. Lung volumes and forced ventilatory flows. Report Working Party Standardization of Lung Function Tests, European Community for Steel and Coal. Official Statement of the European Respiratory Society. Eur Respir J. 1993;16: 5–40.

14. R Development Core Team. R: A language and environment for statistical computing [Internet]. Vienna, Austria: R Foundation for Statistical Computing; 2014. Available:

102 http://www.r-project.org/
