# Supplementary material for: Applicability of the Global Lung Initiative 2012 Reference Values for Spirometry for Longitudinal Data of Elderly Women
Source: PLoS One. 2016 Jun 16;11(6):e0157569. doi: 10.1371/journal.pone.0157569 (PMC4910985; doi:10.1371/journal.pone.0157569)
Supplement: S1 Table — Two one-sided tests for equivalence were performed to establish equivalence between the mean z-score of the GLI reference population and SALIA (p<0.05). (PDF) [file pone.0157569.s003.pdf]

S1 Table. GLI-based z-scores of all healthy never-smoking women (HNSW) with available lung function measurements at baseline and follow-up grouped by time of examination. Two one-sided tests for equivalence were performed to establish equivalence between the mean z-score of the GLI reference population and SALIA ($p<0.05$).

		**N**	**Mean**	**sd**	**p-value***	**%≤LLN**
**Baseline**	FEV_1	299	-0.11	0.90	<0.001	3.68
	FVC	299	0.07	0.81	<0.001	2.34
	FEV_1/FVC	299	-0.35	0.79	0.001	5.35
**Follow-up**	FEV_1	299	0.22	1.03	<0.001	2.68
	FVC	299	0.45	0.99	0.177	1.67
	FEV_1/FVC	299	-0.45	0.81	0.150	8.03

FEV_1: forced expiratory volume in 1 s; FVC: forced vital capacity; LLN: Lower Limit of Normal.

***:** Two one-sided tests (TOST) for equivalence were performed to test on equivalence between the mean z-scores of the GLI reference population and SALIA. A good fit was reached if the null-hypothesis of a mean z-score outside of the interval [-0.5, 0.5] was rejected at the 5% significance level (H_0: mean $z \notin [-0.5,0.5]$).
