# Supplementary material for: Applicability of the Global Lung Initiative 2012 Reference Values for Spirometry for Longitudinal Data of Elderly Women
Source: PLoS One. 2016 Jun 16;11(6):e0157569. doi: 10.1371/journal.pone.0157569 (PMC4910985; doi:10.1371/journal.pone.0157569)
Supplement: S2 Table — Two one-sided tests for equivalence were performed to establish equivalence between the mean z-score of the GLI reference population and SALIA (p<0.05). (PDF) [file pone.0157569.s004.pdf]

S2 Table. GLI-based z-scores of all healthy never-smoking women (HNSW) without symptoms of chronic bronchitis (cough and phlegm for ≥3 months of the year for ≥2 years), chronic cough and chronic phlegm grouped by time of examination (baseline and follow-up). Two one-sided tests for equivalence were performed to establish equivalence between the mean z-score of the GLI reference population and SALIA ($p<0.05$).

		**N**	**Mean**	**sd**	**p-value***	**%≤LLN**
**Baseline**	FEV_1	1631	-0.39	1.05	0.000	11.16
	FVC	1629	-0.19	0.97	0.000	6.51
	FEV_1/FVC	1627	-0.39	0.87	0.000	7.44
**Follow-up**	FEV_1	346	0.20	1.03	0.000	2.89
	FVC	346	0.38	0.96	0.030	2.31
	FEV_1/FVC	346	-0.39	0.84	0.024	6.94

FEV_1: forced expiratory volume in 1 s; FVC: forced vital capacity; LLN: Lower Limit of Normal.

***:** Two one-sided tests (TOST) for equivalence were performed to test on equivalence between the mean z-scores of the GLI reference population and SALIA. A good fit was reached if the null-hypothesis of a mean z-score outside of the interval [-0.5, 0.5] was rejected at the 5% significance level (H_0: mean $z \notin [-0.5, 0.5]$).
