# Supplementary material for: Applicability of the Global Lung Initiative 2012 Reference Values for Spirometry for Longitudinal Data of Elderly Women
Source: PLoS One. 2016 Jun 16;11(6):e0157569. doi: 10.1371/journal.pone.0157569 (PMC4910985; doi:10.1371/journal.pone.0157569)
Supplement: S4 Table — Two one-sided tests for equivalence were performed to establish equivalence between the mean z-score of the GLI reference population and SALIA (p<0.05). (PDF) [file pone.0157569.s006.pdf]

S4 Table. GLI-based z-scores of all healthy never-smoking women (HNSW) who performed the spirometric measurement with MasterScope Jaeger grouped by time of examination (baseline and follow-up). Two one-sided tests for equivalence were performed to establish equivalence between the mean z-score of the GLI reference population and SALIA ($p<0.05$).

		N	Mean	sd	p-value*	%≤LLN
**Baseline**	FEV_1	648	-0.07	0.97	<0.001	4.78
	FVC	649	0.04	0.89	<0.001	3.08
	FEV_1/FVC	648	-0.23	0.88	<0.001	6.17
**Follow-up**	FEV_1	314	0.17	1.04	<0.001	3.18
	FVC	314	0.42	1.01	0.129	1.91
	FEV_1/FVC	314	-0.51	0.78	0.540	7.96

FEV_1: forced expiratory volume in 1 s; FVC: forced vital capacity; LLN: Lower Limit of Normal.

***:** Two one-sided tests (TOST) for equivalence were performed to test on equivalence between the mean z-scores of the GLI reference population and SALIA. A good fit was reached if the null-hypothesis of a mean z-score outside of the interval [-0.5, 0.5] was rejected at the 5% significance level (H_0: mean $z \notin [-0.5, 0.5]$).
