# Supplementary material for: Applicability of the Global Lung Initiative 2012 Reference Values for Spirometry for Longitudinal Data of Elderly Women
Source: PLoS One. 2016 Jun 16;11(6):e0157569. doi: 10.1371/journal.pone.0157569 (PMC4910985; doi:10.1371/journal.pone.0157569)
Supplement: S5 Table — Difference between z-scores at baseline and follow-up (zf − zb). Two one-sided tests for equivalence were performed to establish equivalence between the mean z-scores of the GLI reference population and SALIA. (PDF) [file pone.0157569.s007.pdf]

S5 Table. The longitudinal validity of the GLI reference values of all healthy never-smoking women (HNSW) who performed the spirometric measurement with MasterScope Jaeger. Difference between z-scores at baseline and follow-up ($z_f - z_b$). Two one-sided tests for equivalence were performed to establish equivalence between the mean z-scores of the GLI reference population and SALIA.

**Follow-up - baseline**	**N**	**Mean**	**sd**	**p-value***
FEV_1	132	0.15	0.69	<0.001
FVC	132	0.24	0.68	<0.001
FEV_1/FVC	132	-0.17	0.84	<0.001

FEV_1: forced expiratory volume in 1 s; FVC: forced vital capacity; LLN: Lower Limit of Normal. *: Two one-sided tests (TOST) for equivalence were performed to test on equivalence between the mean deviations in z-scores of the GLI reference population and SALIA. A good longitudinal fit was reached if the null-hypothesis of the mean deviations in z-scores outside of the interval [-0.5, 0.5] was rejected at the 5% significance level (H_0: $z_i - z_j \notin [-0.5, 0.5]$).
